# The Role of a Colon-in-Continuity in Short Bowel Syndrome

**DOI:** 10.3390/nu15030628

**Published:** 2023-01-26

**Authors:** Astrid Verbiest, Palle Bekker Jeppesen, Francisca Joly, Tim Vanuytsel

**Affiliations:** 1 Translational Research Center for Gastrointestinal Disorders (TARGID), Department of Chronic Diseases and Metabolism (ChroMetA), University of Leuven, 3000 Leuven, Belgium; 2 Leuven Intestinal Failure and Transplantation Center (LIFT), University Hospitals Leuven, 3000 Leuven, Belgium; 3 Department of Intestinal Failure and Liver Diseases, Rigshospitalet, Copenhagen University Hospital, 2100 Copenhagen, Denmark; 4 Department of Gastroenterology, IBD and Nutritional Support, Centre for Intestinal Failure and Intestinal Stroke Center, Hôpital Beaujon, UMR 1149, INSERM, University of Paris, 92110 Clichy, France

**Keywords:** short bowel syndrome, intestinal failure, parenteral support, colon-in-continuity, intestinal adaptation, glucagon-like peptide-2 analogs

## Abstract

Short bowel syndrome (SBS) is a rare gastrointestinal condition that is defined as having less than 200 cm of remaining small intestine. SBS results from extensive surgical resection and is associated with a high risk for intestinal failure (IF) with a need for parenteral support (PS). Depending on the region of intestinal resection, three different main anatomy types can be distinguished from each other. In this review, we synthesize the current knowledge on the role of the colon in the setting of SBS-IF with a colon-in-continuity (SBS-IF-CiC), e.g., by enhancing the degree of intestinal adaptation, energy salvage, and the role of the microbiota. In addition, the effect of the disease-modifying treatment with glucagon-like peptide-2 (GLP-2) analogs in SBS-IF-CiC and how it differs from patients without a colon will be discussed. Overall, the findings explained in this review highlight the importance of preservation of the colon in SBS-IF.

## 1. Introduction

### 1.1. Short Bowel Syndrome and Intestinal Failure with a Colon-in-Continuity

**Figure 1:**
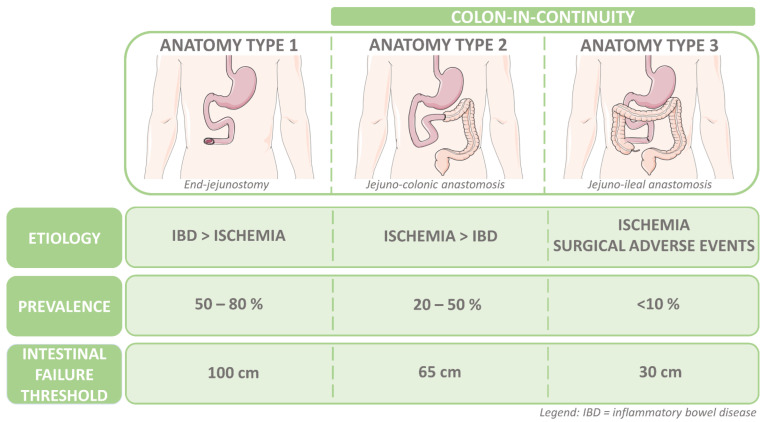
Short Bowel Syndrome Main Anatomy Types in relation to Etiology, Prevalence, and Intestinal Failure Threshold Length. Characterization based on region of intestinal resection. Intestinal failure threshold length relates to the remaining length of small intestine within the three main anatomy types. Adapted from Verbiest et al. [3].

Short bowel syndrome (SBS) is a rare gastrointestinal condition that is defined by a remaining small intestinal length of less than 200 cm as measured from the duodenojejunal junction. SBS usually results from extensive surgical resections, with mesenteric ischemia or inflammatory bowel disease (IBD) as the main underlying causes, or in rare cases, from congenital diseases such as intestinal malformations or gastroschisis [1,2]. Based on the region of intestinal resection, three main anatomical SBS types can be distinguished from each other (Figure 1).

**Figure 2:**
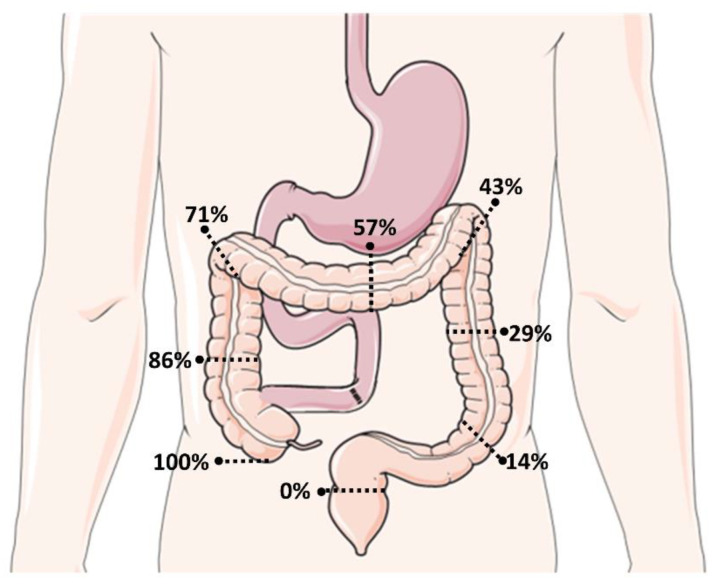
Short Bowel Syndrome with Colon-in-Continuity: preserved colon expressed as % based on Cummings Classification [6].

Anatomy type 1, or the end-jejunostomy, results from a resection of both the ileum and the colon (prevalence: 50–80%). When all or most of the ileum is resected but part of the colon is preserved, this is referred to as a type 2 anatomy or the jejuno-colonic anastomosis (prevalence: 20–50%). Anatomy type 3, or the jejuno-ileal anastomosis, is defined by a remnant of the terminal ileum of at least 10 cm that is in continuity with an intact colon (prevalence: <10%) [4,5]. Patients with either anatomy type 2 or 3 are also referred to as patients with SBS with a colon-in-continuity (SBS-CiC). The part of the colon that is preserved is usually expressed as a percentage according to the Cummings classification (Figure 2) [6].

Among other pathophysiological features, the anatomical changes in SBS interfere with the intestine’s main functions of digestion and absorption because of the reduced intestinal absorptive surface area. The insufficient small intestinal length of patients with SBS is associated with a high risk for the development of both intestinal insufficiency (II) or intestinal failure (IF) depending on the degree of malabsorption. The ESPEN consensus definition defines IF as a loss of gut function that results in a decreased absorption of macronutrients and/or water and electrolytes, for which intravenous supplementation is needed to maintain health and/or growth [1,2]. In contrast to patients with SBS-II who can compensate for malabsorption by increasing oral food intake or using pharmacotherapeutics such as antimotility agents, which reduce the transit time of chyme by increasing non-propulsive segmental motility and reducing antegrade motility [1,7,8,9], patients with SBS-IF are dependent on parenteral support (PS) of nutrients, fluids, and electrolytes for their survival [1]. Metabolic balance studies (MBS) remain the gold standard to discriminate between II and IF based on intestinal absorptive capacity [10]. While the length of the remaining small bowel is of high importance in terms of post-surgical outcome, the colon can be removed without major consequences if the small bowel is intact. This explains why surgeons tend to focus less on the preservation of the colon [11]. However, accumulating research shows that not only the small bowel but also the colon plays an important role in the post-surgical setting in the case of jejuno-colonic anastomosis restoration, especially in reducing the need for PS and ultimately in increasing survival and quality of life [12,13,14]. In the current review, we will discuss the importance of a preserved colon in SBS.

### 1.2. The Functional Characteristics of a Colon in Health

The human large intestine comprises the cecum, colon, and rectum, with a length of approximately 1.5 m. The colon itself can be divided into four anatomical regions: the ascending colon, the transverse colon, the descending colon, and the sigmoid colon. Chyme enters the large intestine via the ileocecal valve (ICV), which connects the cecum to the ileum. The chyme that enters the colon contains only a limited amount of macronutrients (<10%) and more than 80% of water has already been absorbed by the small intestine. What remains are water, electrolytes such as sodium (Na^+^), potassium (K^+^), magnesium (Mg^2+^), and chloride (Cl^−^), and nutrients such as indigestible carbohydrates (i.e., fibers) and, in smaller amounts, lipids and proteins.

The gastrointestinal microbiota contributes to physiological gastrointestinal functions ranging from digestion and absorption of ingested nutrients to the production of micronutrients such as vitamin K and folate to maintaining the immunological intestinal barrier. Microbiota counts vary along the gastrointestinal tract, ranging from 10^4^ colony-forming units/mL in the proximal small bowel to between 10^7^/mL and 10^9^/mL in the terminal ileum to between 10^10^/mL and 10^12^/mL in the colon [15]. While the small bowel comprises an aerobic flora, the colonic microbiota predominantly consists of an anaerobic flora with *Bacteroides*, *Bifidobacterium,* and *Clostridium* as the most prominent species [15,16]. The colonic bacteria will transform indigestible carbohydrates, including oligosaccharides, resistant starch, and soluble non-starch polysaccharides, into gas (including H_2_, CO_2_, and methane) and short-chain fatty acids (SCFA) by saccharolytic fermentation [17]. The main SCFA that are produced are acetate, propionate, and butyrate. About 95% of the produced SCFA are absorbed by the colon, where they will be metabolized and used by the colonocytes as a local energy source [18]. In the distal colon, there is a shift from saccharolytic to proteolytic fermentation by the prevailing microbiota in the case of carbohydrate depletion. Proteolytic fermentation also results in the production of SCFA, together with branched-chain fatty acids [19,20]. Depending on dietary habits, about 6 to 18 g of unabsorbed protein will reach the colon on a daily basis [21].

The colonic epithelial surface is flat with crypts without villi, in contrast to the small intestine, and serves as a barrier against detrimental luminal substances and compounds derived from food and the microbiota. The colonic epithelium consists of three main cell types: columnar, mucous, and enterochromaffin cells. Cells located at the base of the crypts show a high proliferation activity with only a few differentiation markers, whereas the surface cells have a lower tendency to proliferate but show increased levels of differentiation. At the colonic epithelial level, both surface and crypt cells are capable of secreting mucus and absorbing water and electrolytes from the high osmolar chyme [22]. More specifically, the colon is a net absorber of Na^+^ and Cl^−^ and a net secretor of K^+^ and bicarbonate (HCO_3_^−^), and has an absorptive capacity of water up to 4 to 5 L per day [23,24]. The main driver behind water absorption is Na^+^ absorption, which is facilitated by SCFA, inhibited by amiloride, and stimulated by aldosterone [25]. While being mixed with mucus and microbiota, the chyme is transported throughout the colon by colonic mass movements resulting from high-amplitude, propagating contractions. While the ascending and transverse colon mainly play a role in the fermentation and absorption of nutrients, water, and electrolytes from the chyme, the descending and sigmoid colon store the indigestible remnants in either liquid or solid form and gas before propulsion to the rectum and finally defecation or flatulence [24].

## 2. The Role of the Colon in Intestinal Adaptation

### 2.1. Intestinal Adaptation

**Figure 3:**
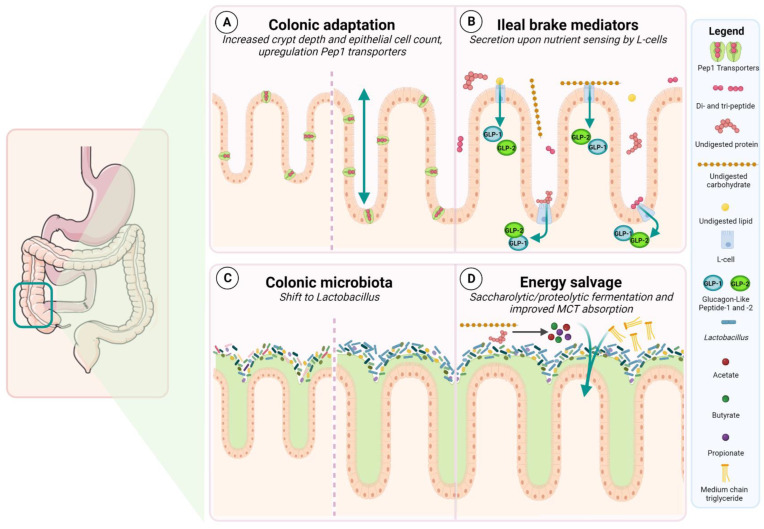
Physiological changes of a preserved colon in continuity in Short Bowel Syndrome with Intestinal Failure. (**A**) Increases in crypt depth and epithelial cell count, and an upregulation of Pep1 transporters are part of the colonic adaptation that takes place within the first two years after extensive surgery. (**B**) L-cells located in the distal ileum and proximal colon secrete GLP-1 and GLP-2 based on nutrient entry in the gut lumen. Both are mediators of the ileal brake and expression is increased when the colon is in continuity. (**C**) Qualitative and quantitative changes of the microbial flora take place when the colon is in continuity: the colonic microbiota of patients with SBS is mainly comprised of *Lactobacillus*. (**D**) The colon serves as an energy-salvaging organ: high levels of undigested carbohydrates and proteins will reach the colon and will be fermented by the microbiota resulting in SCFA production. In addition, MCT absorption is improved. This figure was created with BioRender.com.

Intestinal adaptation is a spontaneous feature that largely takes place within the first two years following extensive surgical resection as a mechanism to compensate for the reduced small intestinal length. By adapting, the remaining intestine is able to absorb nutrients, fluids, and electrolytes more efficiently, resulting in decreased PS requirements. Overall, intestinal adaptation is highly variable in patients with SBS as it is dependent on the underlying pathology, the unresected anatomical sections, and the length of the remnant small bowel [3,14]. Intestinal adaptation can be both structural and/or functional [3]. The improved nutrient and fluid absorption are classically attributed to structural adaptation through the growth of the intestinal villi and deepening of the intestinal crypts (Figure 3A).

Next to the increased absorptive area, structural adaptation also comprises bowel dilatation and lengthening, increased angiogenesis, and increased apoptosis. The functional adaptation is based on a deceleration of the accelerated gastrointestinal transit and alterations in epithelial transporters. To date, evidence on these features and their contribution to human intestinal adaptation after resection remains limited and is mainly based on animal studies (recently reviewed in [3]).

### 2.2. The Role of Food Intake and Hyperphagia

Intestinal absorption and adaptation can be promoted by food intake, both orally and enterally, which improves patient outcomes and reduces the need for PS. Research from Joly et al. showed that continuous enteral feeding in the post-resection setting, either combined with or without oral intake, led to a significant increase in the net absorption of lipids, proteins, and energy compared to oral intake alone [26]. However, oral food intake should also be encouraged. Indeed, hyperphagia, a clinical manifestation that is usually defined as an oral intake 1.5 times greater than a patient’s resting energy expenditure, is reported by 70% of adult patients with SBS [27]. Hyperphagia should also be seen as a compensatory mechanism for malabsorption [28]. This behavioral adaptation contributes to the nutrient passage in the gut and therefore indirectly stimulates the structural and functional adaptation of the remaining intestinal mucosa [27,28,29]. Hyperphagia is reported in patients with SBS, independent of their anatomy type [28].

### 2.3. Why Anatomy Matters

The PS dependence in patients with SBS-IF is influenced by the length of the remnant small bowel and the level of the anastomosis. Messing et al. investigated the PS weaning probabilities in 124 patients with non-malignant SBS-IF receiving home PS [13]. Patients were included if the post-duodenal remnant small intestinal length was ≤150 cm and were followed for up to 16 years. They identified thresholds for permanent IF based on the remnant small intestinal length according to the anatomy type. Most patients with SBS with a small intestinal length of less than 100 cm, 65 cm, and 30 cm in anatomy types 1, 2, and 3, respectively, had a permanent IF probability (Figure 1). Furthermore, the presence of the colon was significantly related with a decreased PS dependency probability, reaching a plateau after 2 years, and an ultimate dependency of 39% in SBS-IF-CiC after 5 years, compared to a dependency of 78% in patients with an end-jejunostomy. This shows that the chance at weaning off PS is significantly related to the presence of at least a part of the colon in continuity (anatomy types 2 and 3). These findings have later been confirmed with a remnant small bowel length of more than 75 cm, the presence of a large part of the colon, and post-operative plasma citrulline levels of at least 20 μmol/L, all of which are associated with a higher likelihood of weaning off PS [12]. Early post-operative measurements of fasting citrulline levels, a non-essential amino acid uniquely produced by the enterocytes of the small intestine, were shown to be a significant marker for home PS dependence. In both cohorts, home PS was permanent in +/− 50% of patients with SBS due to irreversible IF [12,13]. A cutoff at 2 years after resection can reasonably discriminate between transient and permanent intestinal failure, as 95% of patients with transient intestinal failure were able to wean off PS within 24 months. This was in contrast to 94% of patients who did not wean off within 2 years and ultimately were classified as having permanent intestinal failure [12].

Messing et al. also reported a better PS weaning probability in patients with SBS-IF-CiC in whom the cecum and ICV was preserved [13]. The ICV functions as a sphincter-like valve that is responsible for the gradual release of chyme from the ileum into the cecum. The ICV plays an important role in the ileal braking mechanism, which is a distal-to-proximal inhibitory feedback mechanism that controls the release of a meal throughout the gastrointestinal tract to improve nutrient digestion and absorption [30]. Fich et al. showed that the ICV is of lesser importance in regulating ileocolonic transit in healthy individuals, as no differences were observed for small bowel transit or colonic filling when compared to patients with colonic carcinoma who underwent a right hemicolectomy [31]. This finding is in clear contrast to patients with SBS. Nightingale et al. investigated gastric emptying and transit time in 12 healthy volunteers and 13 patients with SBS (7 end-jejunostomies, 6 jejuno-colonic anastomoses) and found that preservation of the colon provided a braking mechanism for the rapid early gastric emptying, which resulted in a normal transit time and allowed for a longer time for liquid absorption [32]. The absence of such a brake contributes to high stomal losses in patients with SBS and an end-jejunostomy. The preservation of the terminal ileum and proximal colon is also related to enteral neuro-hormonal stimuli involved as mediators of the ileal brake [33]. Levels of peptide YY (PYY) were investigated in 12 healthy volunteers and 13 patients with SBS (7 end-jejunostomies, 6 jejuno-colonic anastomoses): while PYY concentration was low in the end-jejunostomy group, higher levels were reported in the SBS-CiC group, which were thought to contribute to the deceleration of gastric emptying and the ileal braking mechanism. Besides PYY, glucagon-like peptide-1 (GLP-1) and -2 (GLP-2) also have a potential role in the ileal brake. These hormones are derived from proglucagon and secreted by L-cells located in the terminal ileum and proximal colon in a 1:1 ratio [34,35]. Nutrient entry in the gut lumen is the primary stimulus for GLP-1 and GLP-2 secretion. However, due to the distal location of L cells, the initial and rapid post-prandial rise in plasma levels is mediated through neural and endocrine stimuli. After a meal, depending on the size and nutrient content, the rise in GLP-1 and GLP-2 levels can be 2- to 5-fold [36,37]. GLP-1 is an incretin hormone that carries out various functions, ranging from stimulating post-prandial insulin release [38] and somatostatin release to inhibiting glucagon secretion [39]. Furthermore, it increases satiety given its anorexigenic signaling [40]. GLP-1 also functions at the gastrointestinal level as it decelerates gastric emptying and intestinal transit [41]. GLP-2, on the other hand, is an intestinotrophic hormone that is involved in the regulation of intestinal energy absorption as well as the maintenance of mucosal morphology and integrity of the intestinal epithelium [41]. By stimulating enterocyte growth, decelerating gastric emptying, and increasing intestinal blood flow, GLP-2 plays a key role in intestinal adaptation [42,43,44,45]. GLP-1 and GLP-2 secretion increases after resection when the distal small bowel and the colon are in continuity (Figure 3B) [46]. Furthermore, the post-prandial increase in plasma GLP-2 levels is associated with the degree of intestinal adaptation [47,48]. This is in stark contrast to patients with an end-jejunostomy who demonstrate no post-prandial increase in GLP-2 given the absence of L-cells [49].

Another enterohormone secreted by the ileum is fibroblast growth factor 19 (FGF19). Binding of bile salts (BS) to the farnesoid X receptor (FXR), located in the terminal ileum, results in the secretion of FGF19 [50]. FGF19-mediated repression of cholesterol 7 α-hydroxylase (CYP7A1) in the liver leads to a suppression of BS synthesis. In patients with SBS with an end-jejunostomy, this negative feedback regulation is absent. Mutanen et al. showed that serum FGF19 levels are low in ileal-resected pediatric patients and that this is associated with liver inflammation and fibrosis [51]. Consequently, increased synthesis of BS will occur, contributing to high jejunostomy outputs. In addition, the stimulated production of BS also plays an important role in the development of intestinal failure-associated liver disease (IFALD), which is a major cause of morbidity in patients with SBS-IF on PS [50]. Analogs of FGF19 are currently in development for patients with nonalcoholic steatohepatitis and could be hypothesized to be beneficial for patients with SBS who underwent ileo-cecal resection too [52].

### 2.4. Energetic Recovery and Restoration of the Fluid and Electrolyte Balance

Restoration of the intestinal continuity by anastomosing the remaining jejunum to the colon resulted in PS cessation in 77% of patients within 5 years [53]. Restoring intestinal continuity also stimulates colonic adaptations, including increases in crypt depth and colonic epithelial cell counts (Figure 3A) [54]. Besides epithelial hyperplasia, changes in intestinal nutrient transporters are also observed. PepT1 transporters are responsible for the intestinal absorption of di- and tripeptides. An upregulation of the PepT1 transporters has been demonstrated to be suggestive of the increased luminal uptake of di- and tripeptides in the colon (Figure 3A) [55]. Furthermore, an energetic recovery and a restoration of the fluid and electrolyte balance take place when the colon is in continuity. 

Increased production of SCFA in the colon contributes to the adaptation process when the colon is in continuity. Both intraluminal colonic infusion and intraperitoneal injection of SCFA have been shown to stimulate mucosal proliferation of the jejunal and ileal segments of the small intestine of normal rats [56,57]. Koruda et al. investigated the effect of PS supplemented with SCFA on the adaptation of the jejunum, ileum, and colon after massive bowel resection in rats [58]. In this study, SCFA supplementation resulted in maintained mucosal morphology in contrast to animals that were fed with regular PS, in which significant jejunal and ileal atrophy was observed. The authors suggested that SCFA may facilitate intestinal adaptation following extensive small bowel resection, but evidence on the effect of SCFA supplementation directly or through microbial changes in humans is still lacking.

The reduced mucosal surface for nutrient and fluid absorption leads to increased amounts of undigested nutrients reaching the colon. In SBS-CiC, the colon serves as an energy-absorbent organ that is capable of increasing the daily SCFA absorption from indigestible carbohydrates (Figure 3D), salvaging up to 1000 kcal per day [59,60,61]. Work from Nordgaard et al. confirmed that the large intestine is crucial in carbohydrate digestion and the salvage of calories in SBS with severe malabsorption [59]. Metabolic balance studies were performed in 8 patients with SBS-CiC and 6 patients with SBS and an end-jejunostomy: A high-carbohydrate low-fat diet reduced fecal energy losses in SBS-CiC when compared to a low-carbohydrate high-fat diet, and energy absorption increased by 20% in the high-carbohydrate diet. In addition, research from Messing et al. also showed that the presence of the colon resulted in greater absorption of carbohydrates when compared to fats and proteins [29].

Norgaard et al. showed that not only carbohydrates but also non-absorbed proteins can be digested by the colon [59,61]. The digestion of proteins reaching the colon is dual: they can either be proteolyzed or fermented by the microbiota. While proteolysis will result in the release of amino acids intended for microbial proliferation, the end products of proteolytic fermentation include SCFA (Figure 3D), branched-chain fatty acids (BCFAs), ammonia, amines, phenols, indoles, and gaseous products such as sulfides [19]. As mentioned earlier, PepT1 transporters are upregulated as part of colonic intestinal adaptation and can be involved in colonic protein salvage [55].

Jeppesen et al. investigated the role of the colon in the absorption of fats, with a focus on medium-chain triglycerides (MCTs) and medium-chain fatty acids (MCFAs) [62]. MCTs are water soluble, contain 8.3 kcal/g, are less dependent on lipase activity, and are more readily absorbed via the portal system, in contrast to long-chain triglycerides (LCT). In total, 19 patients with SBS were included, of which 10 had a preserved colon. Patients were admitted for 9 days to perform a metabolic balance study in which they had to follow either an LCT diet or an LCT + MCT diet in which half of the LCTs were replaced by MCTs in a cross-over fashion. In patients with a preserved colon, the partial replacement of LCTs by MCTs resulted in a considerable improvement in fat absorption and a gain in total energy absorption. These results suggest that MCFAs share a similar ability as SCFA to be absorbed by the colon (Figure 3D).

### 2.5. The Colonic Microbiota in SBS

In SBS-CiC, the microbiota plays an important role in the salvage of energy by the colon [63]. The increased amount of undigested nutrients received by the colon will be fermented by the present flora. The degree of fermentation depends on the retention time of digestion and absorption in relation to the volume of the remaining colon [64]. In addition, the decreased transit time and increased amount of undigested nutrients result in qualitative and quantitative changes in the microbial flora. Massive small-bowel resection causes alterations in the luminal environment, such as an acidic pH and oxygen enrichment, which have an influence on the selective pressures and microbial balance [65,66]. Mucosal and luminal sampling show that the colonic microbiota of patients with SBS is mainly comprised of *Lactobacillus* (Figure 3C). More specifically, the presence of *Lactobacillus mucosae* has been reported to be characteristic of SBS since it was not detected in the flora of a healthy colon [67]. In contrast, species such as *Clostridium leptum* and *Clostridium coccoides,* which are normally present in a healthy colon, were undetectably low or absent in the flora of patients with SBS [63,66,67,68,69]. In addition, Huang et al. suggested that the ICV contributes to distinct microbial features as they showed different intestinal microenvironments between patients with SBS anatomy types 2 and 3, with a greater abundance of *Proteobacteria* in patients with anatomy type 2, whereas the flora of anatomy type 3 was characterized by *Lactobacillus* [65].

Although the adapted microbiota in SBS-CiC plays an important role in the hyperfermentation of undigested nutrients [64], there are also a number of complications in patients with SBS that may result from the altered colonic microbiota and its function, such as small intestinal bacterial overgrowth (SIBO) and D-lactic acidosis. SIBO can be attributed to altered bowel anatomy without compartmentalization (e.g., absence of ICV), altered motility, intestinal acidity, and intestinal dilation [16]. Anaerobic species are primarily responsible for the potentially negative outcomes of SIBO, which include increased intestinal permeability, mucosal inflammation, allergic reactions, and villus atrophy [16,63]. Altogether, SIBO results in nutrient maldigestion, nutrient malabsorption, and deconjugation of bile acids which interfere with fat absorption. Symptoms in patients with SBS that may be attributed to SIBO include anorexia, vomiting, exacerbation of diarrhea and steatorrhea, cramps, bloating, and malnutrition, which all contribute to a decreased quality of life [63].

*Lactobacillus* species are producers of lactic acid, a major end product of carbohydrate fermentation. Lactic acid exists as two stereoisomers: the D- and L-isomers. The difference between both is the orientation around the asymmetric second carbon atom, where the rotation of light in the D-isomer is clockwise and counterclockwise in the L-isomer form. D-2-hydroxy acid dehydrogenase (D-2-HDH), a pH-sensitive enzyme in the liver and kidneys, is of importance in the metabolization of D-lactate [70,71]. In SBS, the colon in continuity, with its altered colonic flora, results in the hypermetabolism of unfermentable carbohydrates, producing substrates such as SCFA and lactate, both D- and L-isomers. The increased amount of organic acids exceeds the metabolization capacity of the colonic microbial flora, leading to an accumulation of acidic products [70]. Both the increased availability of fermentable substrates [72,73] and the acidic environment serve as a growth medium for bacterial species, especially the ones that produce D- and L-lactate, as they are acid resistant [70]. Consequently, the acidic environment serves not only as an initiator of D-lactate production but also as an inhibitor of the metabolization and degradation by D-2-HDH [71] and thus promotes a state of D-lactate acidosis. The central nervous system is predominantly affected by D-lactic acidosis, with symptoms ranging from ataxia, slurred speech, lethargy, and confusion to seizures and coma [16,70]. The diversity of the colonic microbial flora, with different proportions of lactate-producing and lactate-consuming bacteria [68], explains why not all patients with SBS experience D-lactic acidosis. However, clinicians should be aware and suspicious of this phenomenon, with a focus on the fecal D/L lactate ratio as it is a predictor for D-lactate-related neurotoxicity [69].

## 3. GLP-2 Analogs as a Disease Modifying Therapy for SBS-IF and the Effect in SBS-IF-CiC

In the last two decades, GLP-2 and its analogs have sparked pharmacological interest as a potential disease-modifying therapy for SBS-IF. While the exact role of endogenous GLP-2 in spontaneous intestinal adaptation is still not clear, GLP-2 analogs promote an accelerated adaptation or hyperadaptation [3]. Teduglutide (TED) is the first developed and marketed GLP-2 analog for SBS-IF. TED is a short-acting GLP-2 analog with a half-life of 2 up to 3 h requiring daily dosing [44,74,75]. New, chemically modified, longer-acting GLP-2 analogs, glepaglutide and apraglutide, are currently in phase 3 development for the treatment of SBS-IF [76,77,78].

Jeppesen et al. performed an open-label pilot study investigating the safety and efficacy of a 21-day TED treatment with three different doses in patients with SBS with either an end-jejunostomy or at least 50% CiC [79]. Overall, TED was found to be safe and well tolerated, and it significantly reduced intestinal wet weight absorption in all patients. TED significantly increased villus height in patients with an end-jejunostomy. However, TED did not have a significant influence on the crypt depth and mitotic index in the CiC group.

In the pivotal 24-week phase 3 STEPS study, patients with SBS-IF were treated with placebo or TED (0.05 mg/kg) [80]. Overall responder rate (at least 20% PS reduction at weeks 20 and 24) was significantly higher in TED-treated patients (63%) compared to placebo (30%) and 53.8% of TED-treated patients were able to receive at least 1 day off. SBS-IF-CiC responder rates were lower compared to end-jejunostomy patients and failed to reach statistical significance vs. placebo. The long-term safety and efficacy of TED were further investigated in the subsequent 2-year open-label extension STEPS-2 study [81]. PS reductions were observed in all treatment subgroups, with the greatest reductions in patients with the longest TED exposure: after 30 months of TED treatment, 93% of patients had a PS reduction of at least 20%. Moreover, 13 patients achieved enteral autonomy and PS independence. The STEPS-2 study also identified 8 slow responders to TED, of whom 7 were able to achieve a PS reduction of at least 20% in the extension trial. Interestingly, all of these slow responders were CiC patients with remnant small bowel lengths ranging from 30 to 120 cm, with mean PS needs of 12.7 L/week (comparable to the entire STEPS-2 population). These patients had PS reductions between 3.1 and 16.6 L/week over 24 to 104 weeks of TED treatment.

Post hoc analyses have been performed to identify factors associated with the response to TED treatment. While all patients in the STEPS program required >6 months TED treatment in order to achieve enteral autonomy [82], other TED outcomes appeared to be highly variable among the different SBS anatomy types. Reductions in PS were significantly greater in patients who had higher baseline PS needs. These were mainly patients with an end-jejunostomy or ileostomy with IBD as the underlying cause. Furthermore, PS reductions were lower in patients with at least 50% CiC [83]. In general, late TED responders were CiC patients who had a preserved ICV and more colon remaining [84].

More recently, real-world data on TED in SBS-IF in clinical practice have become available and are in line with the earlier observed and described safety and efficacy findings from clinical trials [85,86,87,88]. Interestingly, real-world data suggest that the presence of the colon is a favorable factor in response to GLP-2 analog treatment. In the cohort of Lam et al., 90% of the patients who were able to wean from PS retained at least part of the colon in continuity with variable small bowel lengths [85]. These findings are supported by the French real-world data, which showed that the likelihood of weaning was associated with the presence of a colon in continuity, low baseline PS needs, and a high oral intake [87].

In general, these data suggest that patients with a CiC require a longer treatment period before responding to GLP-2 treatment, which may be related to the different—i.e., higher—endogenous GLP-2 levels in this anatomy subgroup. Moreover, patients with SBS-IF-CiC have a higher likelihood of achieving oral autonomy on GLP-2 analogs. This response profile contrasts with patients with an end-jejunostomy, in whom an early response is observed but with a low probability of full weaning. Potentially, the ideal treatment doses of GLP-2 analogs differ between patients with an end-jejunostomy and CiC, but no data on pharmacokinetic-pharmacodynamic (PK-PD) associations are available in either anatomy group.

## 4. Conclusions

SBS is a rare gastrointestinal condition with a high risk for IF development with a need for PS. The preservation of the colon is associated with several physiological benefits on SBS-IF-CiC outcome. The preservation of the colon has been shown to be beneficial in reducing the need for PS during GLP-2 treatment. This suggests that the colon should be considered an important additional target organ in the treatment of SBS with GLP-2 analogs, or that it is at least an important modifier of the treatment outcome.
